# Multiscale computational analysis of *Xenopus laevis *morphogenesis reveals key insights of systems-level behavior

**DOI:** 10.1186/1752-0509-1-46

**Published:** 2007-10-22

**Authors:** Scott H Robertson, Chris K Smith, Anna L Langhans, Sara E McLinden, Matthew A Oberhardt, Karoly R Jakab, Bette Dzamba, Douglas W DeSimone, Jason A Papin, Shayn M Peirce

**Affiliations:** 1 Department of Biomedical Engineering, University of Virginia, Box 800759, Charlottesville, VA 22908, USA; 2 Department of Cell Biology, University of Virginia, Box 800732, Charlottesville, VA 22908, USA

## Abstract

**Background:**

Tissue morphogenesis is a complex process whereby tissue structures self-assemble by the aggregate behaviors of independently acting cells responding to both intracellular and extracellular cues in their environment. During embryonic development, morphogenesis is particularly important for organizing cells into tissues, and although key regulatory events of this process are well studied in isolation, a number of important systems-level questions remain unanswered. This is due, in part, to a lack of integrative tools that enable the coupling of biological phenomena across spatial and temporal scales. Here, we present a new computational framework that integrates intracellular signaling information with multi-cell behaviors in the context of a spatially heterogeneous tissue environment.

**Results:**

We have developed a computational simulation of mesendoderm migration in the *Xenopus laevis *explant model, which is a well studied biological model of tissue morphogenesis that recapitulates many features of this process during development in humans. The simulation couples, via a JAVA interface, an ordinary differential equation-based mass action kinetics model to compute intracellular Wnt/β-catenin signaling with an agent-based model of mesendoderm migration across a fibronectin extracellular matrix substrate. The emergent cell behaviors in the simulation suggest the following properties of the system: maintaining the integrity of cell-to-cell contact signals is necessary for preventing fractionation of cells as they move, contact with the Fn substrate and the existence of a Fn gradient provides an extracellular feedback loop that governs migration speed, the incorporation of polarity signals is required for cells to migrate in the same direction, and a delicate balance of integrin and cadherin interactions is needed to reproduce experimentally observed migratory behaviors.

**Conclusion:**

Our computational framework couples two different spatial scales in biology: intracellular with multicellular. In our simulation, events at one scale have quantitative and dynamic impact on events at the other scale. This integration enables the testing and identification of key systems-level hypotheses regarding how signaling proteins affect overall tissue-level behavior during morphogenesis in an experimentally verifiable system. Applications of this approach extend to the study of tissue patterning processes that occur during adulthood and disease, such as tumorgenesis and atherogenesis.

## Background

Tissue morphogenesis is a complex biological process that includes the coordinated movements of multiple cells arranging and rearranging in space and time in order to give rise to biological form or structure. Morphogenic processes underpin tissue generation, degeneration, and regeneration during both embryogenesis and adulthood. Understanding the fundamental regulatory mechanisms of tissue morphogenesis has implications for a large array of biological problems, ranging from congenital heart disease [1] to tumorgenesis [2]. Important information about the intracellular signaling processes and cell behaviors underlying morphogenic processes has been amassed in recent decades from reductionist experimental approaches [3], and many of these are currently understood in isolation [4,5]. However, significant questions regarding how cohorts of intra- and intercellular signals orchestrate tissue-level patterning events during morphogenesis remain unanswered because we currently lack the tools needed to understand the translation of biological phenomena across different levels of spatial and temporal scales. The systems-level biology involved in directing cell and tissue rearrangements leading to changes in form has yet to be adequately coupled to mechanisms across length and time scales. This coupling requires the development of new computational approaches and the systematic pairing of these new approaches with cutting-edge experimental studies in model systems that are informative yet tractable.

This paper focuses on modeling a morphogenic process in *Xenopus laevis*, a process that is complex enough to warrant a systems-level approach whose outcome can be highly informative, but one that is simple enough to facilitate a range of targeted experimental manipulations, while still being computationally tractable. Understanding tissue morphogenesis in the *Xenopus laevis *model system is of significant value because the relevant signaling networks and cell behaviors are recapitulated in a number of mammalian developmental and growth processes, such as angiogenesis, wound healing, and tumor growth [6-8]. We have taken a multiscale computational approach for achieving a better understanding of the dynamic interplay between the spatially heterogeneous molecular signals and cellular responses that direct tissue patterning. Our simulation couples a mass action kinetics model of an intracellular signaling pathway, Wnt/β-catenin, with an agent-based model (ABM) of multicell migration and extracellular matrix assembly/degradation to study the process of mesendoderm migration in *Xenopus laevis*.

**Figure 1:**
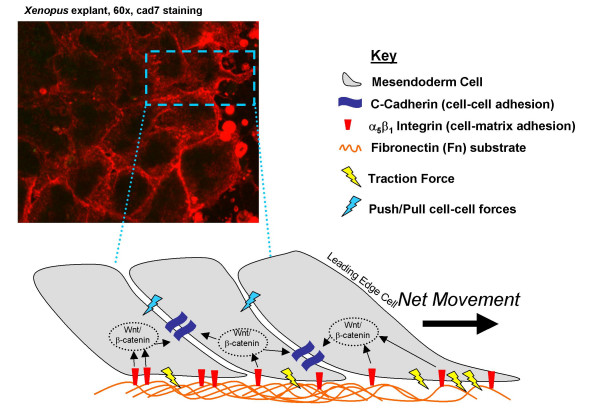
***Xenopus laevis *mesendoderm migration**. (Top Left) Image of *Xenopus laevis *mesendoderm explant, viewed from the top down. (Bottom) Schematic of mesendoderm cell shingling, where cells in the leading edge of the explant overlap the neighboring cells trailing behind them. The key parameters of the multicell model are shown, including: cadherins, integrins, and fibronectin (key located top right).

Mesendoderm migration is the process whereby a circular sheet of cells originating from dorsal, lateral and ventral sides of the embryo traverse hundreds of microns to converge at a common point under the animal cap as gastrulation proceeds [9]. In *Xenopus laevis*, this process occurs between developmental stages 10 and 12, and no other cell population of cells in the embryo migrates as far and as fast as the mesendoderm. Mesendoderm cells give rise to many anterior ventral mesodermal and endodermal structures [4]. From a macroscopic view, mesendoderm migration looks like a wave of cells migrating as a sheet in a uniform direction. However, from a microscopic view, one can see that this process relies on monopolar cell protrusive activity (extension of lamellipodia), dynamic cell-to-cell contacts forming and releasing, cellular arrangement and degradation of the extracellular matrix (ECM), which is composed of fibronectin (Fn), and cells exerting traction forces on the Fn substrate as they move. All of these behaviors are mediated by a cascade of intracellular signaling events occurring within each cell. *Xenopus laevis *mesendoderm migration is easily studied ex vivo by making embryo explants, where a large piece of mesendoderm (encompassing 50+ cells) is surgically excised from the frog embryo and plated on a glass substrate (Figure 1). Explants can be experimentally manipulated to over-express or under-express various proteins and features within cells can be tagged fluorescently enabling their visualization using low-light widefield and confocal microscopy [10,11]. The ease of manipulation and rapid pace at which experiments can be conducted (<3 hours) makes the mesendoderm explant system a particularly appealing biological system in which to study tissue morphogenesis. For this reason, and because many mechanistic details about this process have been well studied in isolation experimentally, mesendoderm migration in the *Xenopus laevis *explant is the focus of the multiscale model herein.

Multiscale computational analysis will inform the field of developmental biology by providing a framework to integrate existing data and to systematically generate and test novel hypotheses to drive experimental design. There are several key remaining challenges to multiscale, systems-level reconstruction and analysis of signaling networks, including: (1) there is a significant lack of intracellular reconstructions for networks implicated in multicellular systems; (2) there is a lack of multicellular models of vertebrate development that capture spatial and temporal heterogeneities in cell phenotype, cell behavior, and tissue environment; (3) there is a need for a computational framework that can integrate across the spatial and temporal scales critical for modeling tissue patterning processes, and determining the computational requirements for obtaining meaningful data across length and time scales is uncharted territory; and (4) rigorous methods for simplifying biological complexities need to be developed and validated, as do routines for setting appropriate boundary conditions in multiscale models. The modeling framework and results presented herein in part address each of these challenges.

Two specific objectives were identified as critical to this modeling effort: (1) the establishment of a computational interface between the two modeling scales (intracellular and multicellular), and (2) a modeling structure that is intuitive and compartmentalizes functionality such that a foundation is provided for future integration of additional intracellular signaling pathways and multicellular behaviors. Consequently, the computational program was organized into sub-functions so additional features could be added without substantially modifying the computer programming code. We present the continuum-based intracellular model and discrete multicellular model separately in the Materials and Methods section. The coupling interface for these two models and its validation is described in the Results section.

## Results and discussion

The development of the multiscale model and associated analyses are summarized below. The intracellular and multicellular models were individually validated to ensure their functionality before combining them into the multiscale model.

### Multi-cellular system model: mesendoderm migration

**Figure 2:**
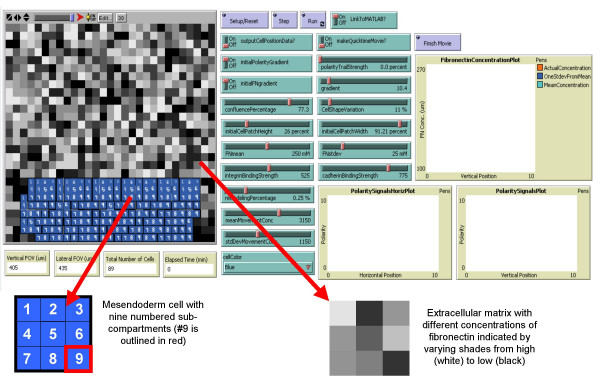
**Multicell ABM simulation environment**. (Top Left) Simulation space where pixels containing Fn are represented by grayscale coloration (bottom right), and nine sub-cellular agents (blue) comprise a single simulated mesendodermal cell (bottom left). (Top Right) Sliders, buttons, and graphical outputs where the user can adjust parameter levels and quantitatively monitor emergent behavior, such as the Fn concentration and accumulation of polarity signals in space and time.

The multicellular system of migrating mesendodermal cells was simulated using an ABM framework, consisting of over 400 two-dimensional (2-D) square agents, each of which occupied a single 2-D pixel at any point in time (Figure 2). The ABM allowed for overlapping agents, or more than one agent to occupy a single 2-D pixel (i.e. enabling "stacking" of agents in the z-direction), such that the ABM approximated the 3-D tissue environment of the *Xenopus laevis *explant model system (Figure 1). The ABM simulated the entire 2.5-hour time span encompassing *in vivo *mesendoderm migration, from developmental stages 10 through 12. Further specific details about the model specific parameters can be found in the Methods section below.

### Modeling sub-cellular compartments as "agents"

In a traditional biological ABM, each biological cell is represented by a single agent [12-15]. However, in this ABM each biological cell was segmented into nine sub-compartments, and each sub-compartment was modeled as a square agent (Figure 2). This sub-cellular resolution enabled different portions of a single cell (e.g. leading edge vs. trailing edge) to respond to different integrin, cadherin, and Fn concentrations. The eight agents that comprise the interfacing regions of the cell (i.e. the outer edges of the cell that are in direct physical contact with neighboring cells) can respond to eight stimuli (left, right, forward, backward, or combinations thereof). The center agent can respond to stimuli without interacting with another agent as well as serve as the "summation" of the stimuli surrounding it. Moreover, the maintenance of cell polarity is important in mesendoderm morphogenesis, and the leading edge of a cell may have experienced a different environment (e.g. cell-cell contact or cell-Fn contact) than the trailing edge of a cell, and these spatial differences would impact the overall behavior of the cell.

Despite the ability to possess different attributes and experience different spatial environments, the sub-cellular agents of a single simulated cell migrated as if they were connected to one another, thus maintaining the overall physical integrity of the simulated biological cell and preventing spatial fractionation or separation of cytoplasmic components. For example, if Agent 1 of Cell 1 resided at the leading edge of a cell sheet, it would extend lamellipodia into the extracellular space, whereas Agent 9 of Cell 1, which resided at the trailing edge of the cell would be in contact with a neighboring trailing cell (e.g. Agent 1 of Cell 2). The ABM simulated 53 independently-acting mesendoderm cells (each represented by 9 "connected" agents) with initial locations in the simulated tissue prescribed according to published fate maps [9]. This number of cells for the grid size described above represents the average density of cells observed in literature [4].

Both *in vivo *and in the *ex vivo *explant model, mesendodermal cells are "shingled" with one another, or spatially juxtaposed, side-by-side and slightly overlapping front-to-back (note the overlapping cells in Figure 2). Shingling is a phenomenon exhibited by mesodermal cells, which enables their collective uni-directional movement. In order to simulate cell shingling in the ABM, we endowed agents with the ability to detect sub-cells on top of them or underneath them and acknowledge their own position in the z-direction as either an overlapping sub-cell or one being overlapped by its neighbor. The extent of shingling was a free parameter that was determined empirically by measuring the extent of shingling in *Xenopus laevis *mesendoderm explants imaged with confocal microscopy. If a cell were entirely on top of another cell, it would have no contact with the Fn substrate rendering it unable to exhibit traction forces on the substrate and preventing migration. Visualizing shingling required that the cells be displayed from trailing end to leading end so that the upper layers stacked on the lower layers.

### Modeling the fibronectin (Fn) extracellular matrix substrate

A protein matrix consisting of Fn fibers provides a substrate for mesendoderm migration, giving migrating cells a structure on which to exert traction forces (i.e., to pull against) during their movement. Mesodermal cell movement has been shown experimentally to require Fn [16], and Fn gradients, in part, are thought to guide the mesendodermal cells as they migrate. Accordingly, the Fn matrix was simulated in the ABM by endowing each 2-D square stationary pixel with a variable, "FnConc", that reflected the relative amount of Fn in each pixel. The amount of Fn in each pixel was represented by gray-scaling the color of each pixel, with white representing the highest concentration and black representing no Fn (note the depiction of Fn in Figure 2). As the simulation ran, sub-cells/agents had the ability to dynamically modify the Fn concentration in each pixel, as described below. Moreover, the Fn concentration in a particular pixel had the ability to impact the behavior of a sub-cell occupying that pixel. For example, cell movement in the ABM was partially dictated by the established hypothesis that cells migrate towards increasing Fn concentrations [17].

### ABM setup

Our ABM framework consisted of a 29 × 27 rectangular grid of 2-D square pixels (783 total pixels, where individual pixels represented 15 *μ*m by 15 *μ*m of tissue). The NetLogo user interface provides a interactive way to set up the initial conditions of the simulation (see Figure 2). A setup function is called by clicking on a "button" in the user interface. "Slider bars", or gauges, containing tick marks allowed the user to prescribe the initial width and length of the simulated embryo explant by setting the slider bars on the desired tick marks. For the simulations presented here, the mesendoderm explant length was set to 71 percent of the total length of the simulation area, or 309 microns, while the width was set to 21 percent, or 85 microns. Note that all simulations were conducted with 53 initial cells defined as squares encompassing a 3 × 3 grid of sub-cellular agents. Each simulated cell encompassed a 45 × 45 micron square area in the simulation space. The initial extent of shingling, or cell overlap could also be prescribed by setting the value of a "slider bar", termed "confluence percentage." For all simulations, the confluence percentage was set to 77%, based on the average empirically-measured value in mesendoderm explant images [4].

Though the necessity of Fn for migration is well documented, there exists no quantitative description of the initial spatial and temporal variations of Fn along the mesendoderm explant. Therefore, we prescribed the initial Fn concentration throughout the 2-D simulation space according to the normal probability distribution:

f(x;μ,σ)=1σ2πe−(x−μ)22σ2
 MathType@MTEF@5@5@+=feaafiart1ev1aaatCvAUfKttLearuWrP9MDH5MBPbIqV92AaeXatLxBI9gBaebbnrfifHhDYfgasaacH8akY=wiFfYdH8Gipec8Eeeu0xXdbba9frFj0=OqFfea0dXdd9vqai=hGuQ8kuc9pgc9s8qqaq=dirpe0xb9q8qiLsFr0=vr0=vr0dc8meaabaqaciaacaGaaeqabaqabeGadaaakeaacqWGMbGzcqGGOaakcqWG4baEcqGG7aWoiiGacqWF8oqBdaWgaaWcbaGaeiilaWcabeaakiab=n8aZjabcMcaPiabg2da9maalaaabaGaeGymaedabaGae83Wdm3aaOaaaeaacqaIYaGmcqWFapaCaSqabaaaaOGaemyzau2aaWbaaSqabeaacqGHsisldaWcaaqaaiabcIcaOiabdIha4jabgkHiTiab=X7aTjabcMcaPmaaCaaameqabaGaeGOmaidaaaWcbaGaeGOmaiJae83Wdm3aaWbaaWqabeaacqaIYaGmaaaaaaaaaaa@4A8F@

where, *μ *= 250 and *σ *= 50, with the Fn concentration within each pixel being randomly assigned a value varying according to this distribution. We also incorporated two "slider bars" that could be used to set the mean and standard deviation of the Fn concentration distribution within the matrix, if the user wished to treat these parameters as free parameters and vary them accordingly.

### ABM parameters

The migratory behaviors of the simulated cells in the ABM were dominated by three key parameters: the relative concentrations of Fn, integrin, and cadherin proteins. As already discussed, Fn is an acellular ECM protein found in the substrate upon which cells migrate. Mesodermal cells bind Fn through α_5_β_1 _integrin, which modulates cell traction forces and, in part, affects cell migration speed [18]. Cell-cell contact forces are imposed as neighboring cells push and pull each other as they migrate on Fn, and these interactions in mesendoderm are mediated by C-cadherin (simply referred to as "cadherin" hereafter). Cadherins are cell membrane-bound proteins that bind to cadherins on neighboring cells and transmit force from one cell to its neighbor [19]. In the ABM, cadherin binding was limited to regions where sub-cells (agents) spatially overlapped (shingled) so that force transmission from cell to cell occurred only when neighbors were in direct contact with one another. Concentrations of engaged/activated cadherins were determined much in the same way that integrin concentrations were determined: according to the percentage of total cell surface area (i.e. the proportion of the 9 sub-cellular agents comprising each cell) physically in contact with a neighboring cell.

Levels of these three parameters differed for each cell and across cells in space and time (depending on their location in the tissue and their connectivity and shingling with other cells) but they were recorded for each cell over time. Initial levels of these parameters for each cell were set by identifying the ratio of cadherins to integrins as observed in literature. Two free parameters controlled the integrin and cadherin binding strength, and these are prescribed by the user adjusting "slider-bars."

As the simulation ran, the Fn concentration sensed by a particular cell was determined by the amount of Fn contained within the pixel underneath it at that time point. In the current ABM, all cells were assumed to have the same basal expression levels of α_5_β_1 _integrin (defined as one "relative unit"). The concentration of activated (Fn-bound) α_5_β_1 _integrin was scaled according to the percentage of cell surface area in contact with Fn, which was dictated by the number of sub-cellular agents in direct contact with the Fn substrate. For example, cells with 30% of their surface area in contact with the Fn substrate would possess an α_5_β_1 _integrin value equal to 0.3. Cadherin concentrations for each cell were determined much in the same way, but based on the surface area in contact with a neighboring cell. As the ABM progressed through time, levels of these variables changed for each cell, depending on the cell's contact with other mesendodermal cells and the extent of its adhesion with the Fn substrate. These ABM rules are delineated in Table 1.

**Table 1:** Parameter values used in the ABM multicellular simulation.

**Parameter Type**	**Parameter (units)**	**Value**	**Reference**
Initial Conditions	Amount of Fn per patch (relative units; prescribed as the mean and standard deviation of the normal distribution)	Mean = 250; S.D. = 50	unpublished observation in *Xenopus laevis *explants
	Initial gradient of Fn (Fn concentration scaling factor for each pixel row that ensures a vertical gradient in the initial Fn concentration; higher values of this parameter generate a steeper gradient)	10	unpublished observation in *Xenopus laevis *explants
	Total number of mesendodermal cells	53	[9]
	Extent of cell shingling (percentage of cell surface area overlapping the neighboring cell behind it)	77	[9]
	Width of explant (microns)	85	[9]
	Length of explant (microns)	309	[9]
	Cadherin activation (mN/m^2^)	2.5	[21]
	Integrin activation (mN/m^2^)	1.5	[21]
Degradation of Fn	Amount of degradation (reduction in Fn as a percentage of previous amount in that pixel prior to degradation by cell)	0.3	unpublished observation in *Xenopus laevis *explants
Polarity Signal	Strength (percent contribution of the polarity signal to the direction or heading of the cell)	4.7	hypothesized based on [25]
Migration	Mean distance traveled per time step (microns)	6.5	[9]

### Quantifying and visualizing simulated mesendoderm behaviors

**Figure 3:**
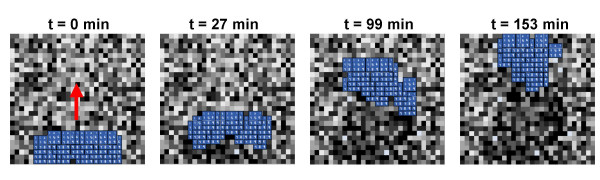
**Screenshots of mesendoderm migration in ABM at different time points**. Blue cells migrate in the direction of the red arrow over the ~2.5 hour time window. Cell degradation of the Fn matrix is visible in the darker pixels left behind as they move forward. Global thinning of the explant in the lateral direction (left to right) and lengthening in the longitudinal direction (top to bottom) is also evident.

Key behaviors that were simulated by the ABM included cell migration across the stationary pixels and modification of the Fn matrix contained within each pixel. The ABM framework recorded these behaviors at each time point by writing the spatial Fn concentrations at each x- and y- pixel coordinate to an output text file and by writing the x and y coordinates of each cell to an output text file. The ABM also visually represented cell movements and Fn concentrations within each pixel (through grayscale pixel coloration scaled to represent the Fn concentration) (Figure 3). A Microsoft Excel macro was used to calculate average cell speeds, as described below.

Each cell in the ABM moved independently from one another, but its movements were impacted by the local Fn concentration of the cell and connectivity/shingling with neighboring cells. Cells were instructed to move generally towards increasing Fn concentrations; however, they were also simultaneously pushed and pulled by neighboring cells. Consequently, each movement calculation required the spatial summation of force vectors from each sub-cell into a net force vector consisting of the forces that cells exerted on Fn and the push/pull forces exerted by neighboring (shingled) cells. Note that individual forces on cells were not balanced, per se, which is a limitation of the current model; however, by using a simple relationship to correlate the net amount of force experienced by a cell with migratory behavior (Eq. 7), the ABM was able to predict realistic migratory behaviors.

The Fn gradient around each sub-cell was calculated by averaging the Fn concentrations in the 8 surrounding pixels. The first part of the movement algorithm calculated the net direction of the gradient by summing the surrounding Fn concentrations in the *x *and *y *directions. The gradient was a function of concentration and *θ*, the angular position of the pixel relative to the subcell (Equations 1 and 2).

FnGradxDirection=∑SurroundingPixel=18(fibronectinConcSurroundingPixel)∗cos⁡(θsurroundingPixel)
 MathType@MTEF@5@5@+=feaafiart1ev1aaatCvAUfKttLearuWrP9MDH5MBPbIqV92AaeXatLxBI9gBaebbnrfifHhDYfgasaacH8akY=wiFfYdH8Gipec8Eeeu0xXdbba9frFj0=OqFfea0dXdd9vqai=hGuQ8kuc9pgc9s8qqaq=dirpe0xb9q8qiLsFr0=vr0=vr0dc8meaabaqaciaacaGaaeqabaqabeGadaaakeaacqWGgbGrcqWGUbGBcqWGhbWrcqWGYbGCcqWGHbqycqWGKbazdaWgaaWcbaGaemiEaGNaemiraqKaemyAaKMaemOCaiNaemyzauMaem4yamMaemiDaqNaemyAaKMaem4Ba8MaemOBa4gabeaakiabg2da9maaqahabaGaeiikaGIaemOzayMaemyAaKMaemOyaiMaemOCaiNaem4Ba8MaemOBa4MaemyzauMaem4yamMaemiDaqNaemyAaKMaemOBa4Maem4qamKaem4Ba8MaemOBa4Maem4yam2aaSbaaSqaaiabdofatjabdwha1jabdkhaYjabdkhaYjabd+gaVjabdwha1jabd6gaUjabdsgaKjabdMgaPjabd6gaUjabdEgaNjabdcfaqjabdMgaPjabdIha4jabdwgaLjabdYgaSbqabaGccqGGPaqkaSqaaiabdofatjabdwha1jabdkhaYjabdkhaYjabd+gaVjabdwha1jabd6gaUjabdsgaKjabdMgaPjabd6gaUjabdEgaNjabdcfaqjabdMgaPjabdIha4jabdwgaLjabdYgaSjabg2da9iabigdaXaqaaiabiIda4aqdcqGHris5aOGaey4fIOIagi4yamMaei4Ba8Maei4CamNaeiikaGccciGae8hUde3aaSbaaSqaaiabdohaZjabdwha1jabdkhaYjabdkhaYjabd+gaVjabdwha1jabd6gaUjabdsgaKjabdMgaPjabd6gaUjabdEgaNjabdcfaqjabdMgaPjabdIha4jabdwgaLjabdYgaSbqabaGccqGGPaqkaaa@A8DD@

FnGradyDirection=∑SurroundingPixel=18(fibronectinConcSurroundingPixel)∗sin⁡(θsurroundingPixel)
 MathType@MTEF@5@5@+=feaafiart1ev1aaatCvAUfKttLearuWrP9MDH5MBPbIqV92AaeXatLxBI9gBaebbnrfifHhDYfgasaacH8akY=wiFfYdH8Gipec8Eeeu0xXdbba9frFj0=OqFfea0dXdd9vqai=hGuQ8kuc9pgc9s8qqaq=dirpe0xb9q8qiLsFr0=vr0=vr0dc8meaabaqaciaacaGaaeqabaqabeGadaaakeaacqWGgbGrcqWGUbGBcqWGhbWrcqWGYbGCcqWGHbqycqWGKbazdaWgaaWcbaGaemyEaKNaemiraqKaemyAaKMaemOCaiNaemyzauMaem4yamMaemiDaqNaemyAaKMaem4Ba8MaemOBa4gabeaakiabg2da9maaqahabaGaeiikaGIaemOzayMaemyAaKMaemOyaiMaemOCaiNaem4Ba8MaemOBa4MaemyzauMaem4yamMaemiDaqNaemyAaKMaemOBa4Maem4qamKaem4Ba8MaemOBa4Maem4yam2aaSbaaSqaaiabdofatjabdwha1jabdkhaYjabdkhaYjabd+gaVjabdwha1jabd6gaUjabdsgaKjabdMgaPjabd6gaUjabdEgaNjabdcfaqjabdMgaPjabdIha4jabdwgaLjabdYgaSbqabaGccqGGPaqkaSqaaiabdofatjabdwha1jabdkhaYjabdkhaYjabd+gaVjabdwha1jabd6gaUjabdsgaKjabdMgaPjabd6gaUjabdEgaNjabdcfaqjabdMgaPjabdIha4jabdwgaLjabdYgaSjabg2da9iabigdaXaqaaiabiIda4aqdcqGHris5aOGaey4fIOIagi4CamNaeiyAaKMaeiOBa4MaeiikaGccciGae8hUde3aaSbaaSqaaiabdohaZjabdwha1jabdkhaYjabdkhaYjabd+gaVjabdwha1jabd6gaUjabdsgaKjabdMgaPjabd6gaUjabdEgaNjabdcfaqjabdMgaPjabdIha4jabdwgaLjabdYgaSbqabaGccqGGPaqkaaa@A8E9@

Every sub-cell agent performed this gradient calculation, so each cell had a total of nine Fn concentration vectors. A net Fn concentration vector was then calculated by summing all of the sub-cell Fn concentration vectors within a given cell, and multiplying by a scaling factor that represented the average binding force of the integrins within that cell (Eqs. 3 and 4):

NetFnConcxDirection=AvergeBindingForce∗∑Subcell=19netSubcellForcexDirection
 MathType@MTEF@5@5@+=feaafiart1ev1aaatCvAUfKttLearuWrP9MDH5MBPbIqV92AaeXatLxBI9gBaebbnrfifHhDYfgasaacH8akY=wiFfYdH8Gipec8Eeeu0xXdbba9frFj0=OqFfea0dXdd9vqai=hGuQ8kuc9pgc9s8qqaq=dirpe0xb9q8qiLsFr0=vr0=vr0dc8meaabaqaciaacaGaaeqabaqabeGadaaakeaacqWGobGtcqWGLbqzcqWG0baDcqWGgbGrcqWGUbGBcqWGdbWqcqWGVbWBcqWGUbGBcqWGJbWydaWgaaWcbaGaemiEaGNaemiraqKaemyAaKMaemOCaiNaemyzauMaem4yamMaemiDaqNaemyAaKMaem4Ba8MaemOBa4gabeaakiabg2da9iabdgeabjabdAha2jabdwgaLjabdkhaYjabdEgaNjabdwgaLjabdkeacjabdMgaPjabd6gaUjabdsgaKjabdMgaPjabd6gaUjabdEgaNjabdAeagjabd+gaVjabdkhaYjabdogaJjabdwgaLjabgEHiQmaaqadabaGaemOBa4MaemyzauMaemiDaqNaem4uamLaemyDauNaemOyaiMaem4yamMaemyzauMaemiBaWMaemiBaWMaemOrayKaem4Ba8MaemOCaiNaem4yamMaemyzau2aaSbaaSqaaiabdIha4jabdseaejabdMgaPjabdkhaYjabdwgaLjabdogaJjabdsha0jabdMgaPjabd+gaVjabd6gaUbqabaaabaGaem4uamLaemyDauNaemOyaiMaem4yamMaemyzauMaemiBaWMaemiBaWMaeyypa0JaeGymaedabaGaeGyoaKdaniabggHiLdaaaa@8F99@

NetFnConcyDirection=AvergeBindingForce∗∑Subcell=19netSubcellForceyDirection
 MathType@MTEF@5@5@+=feaafiart1ev1aaatCvAUfKttLearuWrP9MDH5MBPbIqV92AaeXatLxBI9gBaebbnrfifHhDYfgasaacH8akY=wiFfYdH8Gipec8Eeeu0xXdbba9frFj0=OqFfea0dXdd9vqai=hGuQ8kuc9pgc9s8qqaq=dirpe0xb9q8qiLsFr0=vr0=vr0dc8meaabaqaciaacaGaaeqabaqabeGadaaakeaacqWGobGtcqWGLbqzcqWG0baDcqWGgbGrcqWGUbGBcqWGdbWqcqWGVbWBcqWGUbGBcqWGJbWydaWgaaWcbaGaemyEaKNaemiraqKaemyAaKMaemOCaiNaemyzauMaem4yamMaemiDaqNaemyAaKMaem4Ba8MaemOBa4gabeaakiabg2da9iabdgeabjabdAha2jabdwgaLjabdkhaYjabdEgaNjabdwgaLjabdkeacjabdMgaPjabd6gaUjabdsgaKjabdMgaPjabd6gaUjabdEgaNjabdAeagjabd+gaVjabdkhaYjabdogaJjabdwgaLjabgEHiQmaaqadabaGaemOBa4MaemyzauMaemiDaqNaem4uamLaemyDauNaemOyaiMaem4yamMaemyzauMaemiBaWMaemiBaWMaemOrayKaem4Ba8MaemOCaiNaem4yamMaemyzau2aaSbaaSqaaiabdMha5jabdseaejabdMgaPjabdkhaYjabdwgaLjabdogaJjabdsha0jabdMgaPjabd+gaVjabd6gaUbqabaaabaGaem4uamLaemyDauNaemOyaiMaem4yamMaemyzauMaemiBaWMaemiBaWMaeyypa0JaeGymaedabaGaeGyoaKdaniabggHiLdaaaa@8F9D@

### The importance of cell shingling

The net Fn concentration vector dictated the magnitude and direction of cell movement if the Fn gradient was the only external influence on the cell. Without shingling (and the cadherin-mediated forces exerted by neighboring cells), each sub-cell migrated directly up the local Fn concentration gradient, and the ABM predicted dis-aggregation of the cells in the simulated tissue, which is not physiologically observed. However, by incorporating a shingling (cadherin-mediated force) according to the following function,

shinglingForcecell=∑shingledCells(shingledAreacellArea)∗netForceshingledCell∗cadherinBindingForce
 MathType@MTEF@5@5@+=feaafiart1ev1aaatCvAUfKttLearuWrP9MDH5MBPbIqV92AaeXatLxBI9gBaebbnrfifHhDYfgasaacH8akY=wiFfYdH8Gipec8Eeeu0xXdbba9frFj0=OqFfea0dXdd9vqai=hGuQ8kuc9pgc9s8qqaq=dirpe0xb9q8qiLsFr0=vr0=vr0dc8meaabaqaciaacaGaaeqabaqabeGadaaakeaacqWGZbWCcqWGObaAcqWGPbqAcqWGUbGBcqWGNbWzcqWGSbaBcqWGPbqAcqWGUbGBcqWGNbWzcqWGgbGrcqWGVbWBcqWGYbGCcqWGJbWycqWGLbqzdaWgaaWcbaGaem4yamMaemyzauMaemiBaWMaemiBaWgabeaakiabg2da9maaqafabaWaaeWaaeaadaWcaaqaaiabdohaZjabdIgaOjabdMgaPjabd6gaUjabdEgaNjabdYgaSjabdwgaLjabdsgaKjabdgeabjabdkhaYjabdwgaLjabdggaHbqaaiabdogaJjabdwgaLjabdYgaSjabdYgaSjabdgeabjabdkhaYjabdwgaLjabdggaHbaaaiaawIcacaGLPaaaaSqaaiabdohaZjabdIgaOjabdMgaPjabd6gaUjabdEgaNjabdYgaSjabdwgaLjabdsgaKjabdoeadjabdwgaLjabdYgaSjabdYgaSjabdohaZbqab0GaeyyeIuoakiabgEHiQiabd6gaUjabdwgaLjabdsha0jabdAeagjabd+gaVjabdkhaYjabdogaJjabdwgaLnaaBaaaleaacqWGZbWCcqWGObaAcqWGPbqAcqWGUbGBcqWGNbWzcqWGSbaBcqWGLbqzcqWGKbazcqWGdbWqcqWGLbqzcqWGSbaBcqWGSbaBaeqaaOGaey4fIOIaem4yamMaemyyaeMaemizaqMaemiAaGMaemyzauMaemOCaiNaemyAaKMaemOBa4MaemOqaiKaemyAaKMaemOBa4MaemizaqMaemyAaKMaemOBa4Maem4zaCMaemOrayKaem4Ba8MaemOCaiNaem4yamMaemyzaugaaa@AD33@

the characteristic aggregate movement of the mesendoderm that has been observed experimentally was recovered. By adding the Fn force vector with the shingling force vectors (Eq. 6),

*final Force Vector*_*subcell *_= *fibronectin Vector*_*subcell *_+ *shingling Vector*_*subcell*_

the model derived the net force that each cell experienced, which was directly related to its final movement. The final step in the calculation of the movement was to translate the net force into a distance that the cell traveled. Low forces resulted in smaller movement, and higher forces resulted in migration over larger distances. The model restricted the maximum movement of any cell to be 15 *μ*m, the width of one pixel. This limitation ensured that cells did not completely overtake other cells, which is not observed. The function to translate forces into distances was an exponentially limited normal distribution (Eq. 7).

distanceTraveled={zScore<0ezScore+ln⁡(12)zScore≥01−eln⁡(12)−zScore
 MathType@MTEF@5@5@+=feaafiart1ev1aaatCvAUfKttLearuWrP9MDH5MBPbIqV92AaeXatLxBI9gBaebbnrfifHhDYfgasaacH8akY=wiFfYdH8Gipec8Eeeu0xXdbba9frFj0=OqFfea0dXdd9vqai=hGuQ8kuc9pgc9s8qqaq=dirpe0xb9q8qiLsFr0=vr0=vr0dc8meaabaqaciaacaGaaeqabaqabeGadaaakeaaieGacqWFKbazcqWFPbqAcqWFZbWCcqWF0baDcqWFHbqycqWFUbGBcqWFJbWycqWFLbqzcqWFubavcqWFYbGCcqWFHbqycqWF2bGDcqWFLbqzcqWFSbaBcqWFLbqzcqWFKbazcqGH9aqpdaGabaqaauaabeqaciaaaeaacqWG6bGEcqWGtbWucqWGJbWycqWGVbWBcqWGYbGCcqWGLbqzcqGH8aapcqaIWaamaeaacqWGLbqzdaahaaWcbeqaaiabdQha6jabdofatjabdogaJjabd+gaVjabdkhaYjabdwgaLjabgUcaRiGbcYgaSjabc6gaUnaabmaabaWaaSGaaeaacqaIXaqmaeaacqaIYaGmaaaacaGLOaGaayzkaaaaaaGcbaGaemOEaONaem4uamLaem4yamMaem4Ba8MaemOCaiNaemyzauMaeyyzImRaeGimaadabaGaeGymaeJaeyOeI0Iaemyzau2aaWbaaSqabeaacyGGSbaBcqGGUbGBdaqadaqaamaaliaabaGaeGymaedabaGaeGOmaidaaaGaayjkaiaawMcaaiabgkHiTiabdQha6jabdofatjabdogaJjabd+gaVjabdkhaYjabdwgaLbaaaaaakiaawUhaaaaa@7CAF@

The exponentially limited normal distribution was used because it is a smooth function, with upper and lower limits of 0 *μ*m and 13 *μ*m, respectively. The equation was an approximation to the cellular response and was justified only by qualitative data, thereby making it a potential source of error. In order to compensate for the potential inaccuracy, the mean distance traveled (set to 6.5 *μ*m for the initial simulation), could be set to correspond to any force value. Likewise, the standard deviation of the distance traveled is also a free parameter that can be determined empirically.

Once all of the cells calculated a distance and direction, the movement occurred. All nine of the sub-cells moved the same distance in the same direction, which ensured that no cell (agent) fractionated into pieces. Cells at the leading edge moved first, followed by cells behind them.

### The importance of Fn

**Figure 4:**
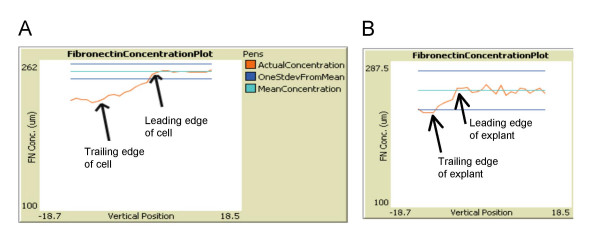
**Calculating cell force vectors from fibronectin gradients**. (A) Fn concentration across a single cell. As cells move forward, a gradient of fibronectin is established from the leading edge to the trailing edge. (B) A Fn gradient is also established across the entire explant length.

When mesendoderm cells migrate on the Fn substrate, their integrins pull on the Fn matrix, which can cause structural changes that modify the ability for other cells to bind Fn, thereby affecting cell migration of trailing cells. To model this matrix rearrangement (e.g., degradation), every sub cell reduced the amount of usable Fn each time it migrated over a pixel. The extent of Fn degradation by cells was a free parameter that was set using a "slider bar" to a value that was determined empirically. The value was set to 0.3% (the reduction of the Fn concentration under one cell after one time step). This value will need to be refined in future iterations of the model. However, empirically it was determined that a value much higher resulted in the leading edge consuming so much Fn that the trailing edge did not have matrix on which to migrate; too small of a value resulted in random movement of the cells. Local Fn gradients were established from the leading (higher concentration) to the trailing edge (lower concentration) of a cell (Figure 4A). As the cells moved forward, a global Fn gradient in the explant tissue was established since the Fn matrix at the trailing edge was more degraded than the Fn matrix at the leading edge (Figure 4B).

We hypothesized that the Fn gradient was one of the major driving forces behind the collective movement of the migrating cells. Because cells moved towards increasing Fn concentrations, the establishment of a gradient served as a biasing force to direct the cells in a uniform direction. Thus, the migration of cells created a Fn concentration gradient, which sustained the forward movement. This self-sustainability suggests that natural feedback mechanisms within mesendodermal cells create systematic movement from randomness [4]. However, by implementing an initial Fn gradient at the outset of the simulation, the cells moved at a faster rate because they did not have to wait for the gradient to evolve. This result suggests a method for the multicellular system to implement a positive feedback process as the matrix serves as a signal from one cell to another.

### Consistent model system behaviors

The initial testing of the multicellular model was qualitative to ensure that the characteristic motion of the migrating cells was accurate. Specifically, three key emergent phenomena were necessary to prove basic movement accuracy: forward movement, collective movement, and shingling. In the preliminary tests, however, the cells did not behave as expected. Rather than moving towards a common location as a cohesive unit, the cells scattered in multiple directions and exhibited only limited shingling. The inaccuracies suggested the model was incomplete. After reviewing the code and running multiple simulations, it was determined that the modeled cell behavior did not have a sufficient accounting of polarity.

### Importance of polarity signals

Polarity was incorporated into the multicellular simulation in an attempt to address the shortcoming described above. The term "polarity signal" refers to structural and/or biochemical rearrangements of the Fn matrix by migrating cells [20]. These changes provide directional information on past cell movements (e.g., "ruts" define the most traveled areas of a road). In the multicellular model, cells were programmed to leave a polarity signal on each pixel they occupied, which biased the migration of future cells towards that pixel. The results of multiple simulations suggested that the polarity signals were necessary for cells to move forward and towards one another, causing them to cluster and shingle in a way that is qualitatively consistent with what has been observed experimentally (Figure 3). This result suggests that polarity signals are not only relevant to mesendoderm migration, but they may be essential in this morphogenic process. There are certainly other mechanisms for cell polarity in this process such as gradients of diffusible signals that are not yet accounted for in this model but may be the subject of further improvements as discussed below. In addition, other mechanisms for collective movement will need to be explored as the model is expanded and refined.

### Importance of cadherin-integrin balance

In addition to the "polarity signal," a balance between cadherin and integrin-mediated forces was necessary to ensure accurate aggregate movements. As long as the assigned integrin binding strength was on the same order of magnitude as the cadherin binding strength (~2.5 mN/m^2^), there was sufficient force to keep neighboring cells together. This observation emerged from running multiple simulations and is consistent with what has been observed experimentally [21]. This balance of cell-cell and cell-matrix forces emerged from the multicellular simulation presented herein.

### Factors impacting cell migration speed

The average migration speed of mesendodermal cells in the explant model has been reported as 100 *μ*m/hour [4]. The multicellular model was therefore calibrated by running the simulation for ten trials and calculating the average cell movement per frame. The number of frames per hour was then calculated from the average cell movement per frame (Equation 8).

framesPerHour=movementPerFrameaverageCellVelocity
 MathType@MTEF@5@5@+=feaafiart1ev1aaatCvAUfKttLearuWrP9MDH5MBPbIqV92AaeXatLxBI9gBaebbnrfifHhDYfgasaacH8akY=wiFfYdH8Gipec8Eeeu0xXdbba9frFj0=OqFfea0dXdd9vqai=hGuQ8kuc9pgc9s8qqaq=dirpe0xb9q8qiLsFr0=vr0=vr0dc8meaabaqaciaacaGaaeqabaqabeGadaaakeaacqWGMbGzcqWGYbGCcqWGHbqycqWGTbqBcqWGLbqzcqWGZbWCcqWGqbaucqWGLbqzcqWGYbGCcqWGibascqWGVbWBcqWG1bqDcqWGYbGCcqGH9aqpdaWcaaqaaiabd2gaTjabd+gaVjabdAha2jabdwgaLjabd2gaTjabdwgaLjabd6gaUjabdsha0jabdcfaqjabdwgaLjabdkhaYjabdAeagjabdkhaYjabdggaHjabd2gaTjabdwgaLbqaaiabdggaHjabdAha2jabdwgaLjabdkhaYjabdggaHjabdEgaNjabdwgaLjabdoeadjabdwgaLjabdYgaSjabdYgaSjabdAfawjabdwgaLjabdYgaSjabd+gaVjabdogaJjabdMgaPjabdsha0jabdMha5baaaaa@6E46@

This analysis determined that 0.286 simulation time steps in the ABM correlated to one minute, and the time scale of the agent-based model was calibrated so the simulated movements represented accurate cell speeds.

**Figure 5:**
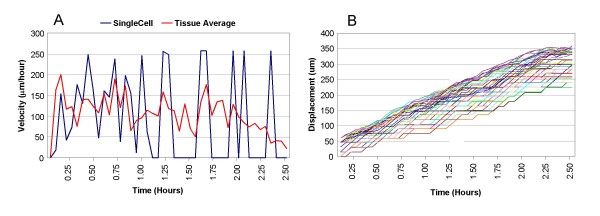
**Velocity and displacement of individual cells and the explant tissue**. (A) Velocity (*μ*m/hr) of single mesendodermal cell (blue) vs. explant average (red). The oscillations in single cell velocity reflect the temporal balance between cadherin and integrin signaling, as well as the Fn gradient under the cell, which is in a gradient across the tissue, but still somewhat random from pixel to pixel. The velocity returns to 0 at the end of the simulation because the leading cell reaches the boundary of the substrate. (B) Displacement (*μ*m) of single cells over time. Each colored tracing represents the displacement of a single cell in the explant, defined as the distance traveled from the cell's initial position in the simulation space.

Migration velocities of individual cells varied greatly and were highly dependent on differences in connectivity with neighboring cells (cadherin activation). The average velocity of the migrating tissue, however, was more dependent on the magnitude of variation within the Fn distribution (Figure 5A). The increased variation among individual cell velocities is hypothesized to be a function of the differing strength of the cell-cell interactions.

### Emergent tissue morphogenesis

The simulated mesendoderm explant tended to narrow laterally and extend vertically in the direction of migration. The initial width of the explant was 85 *μ*m, and after two hours of simulated time, the tissue height doubled (Figure 5B). This thinning and lengthening behavior has been qualitatively observed in the Keller explant model. We hypothesize that the narrowing resulted from shingling forces and polarity signals, both of which encouraged cell aggregation. We hypothesize that the lengthening could be explained by the combined forward migration and narrowing of the tissue. It is important to note that this phenomenon was not programmed or "hard-wired" into the simulation, but emerged from the individual behaviors of the individual cells and sub-cells.

### Integration and validation of multiscale model

There are multiple interactions between the Wnt/β-catenin signaling network and the mesodermal migration behavior described above. It has been hypothesized that the Fn concentration surrounding a cell impacts the strength of the initial Wnt signal [22]. To simulate this interaction, we coupled the intracellular signaling model with the multicell ABM, such that each cell in the ABM was underpinned by its own intracellular signaling model. The multiscale model is, therefore, a multicellular ABM (see Additional File 1) that utilizes a JAVA extension (see Additional File 2) to run Matlab^® ^simulations (see Additional File 3) of the intracellular signaling pathways for each cell at every time step.

NetLogo and Matlab^® ^are not directly compatible, so an intermediary JAVA program was designed to interface the two. NetLogo has extension capabilities that allow JAVA code to be called from within NetLogo code. The JAVA code was a simple JAR file that called Matlab^® ^and executed the intracellular simulation. This connection allowed the two scales to interact dynamically as one multiscale model.

**Figure 6:**
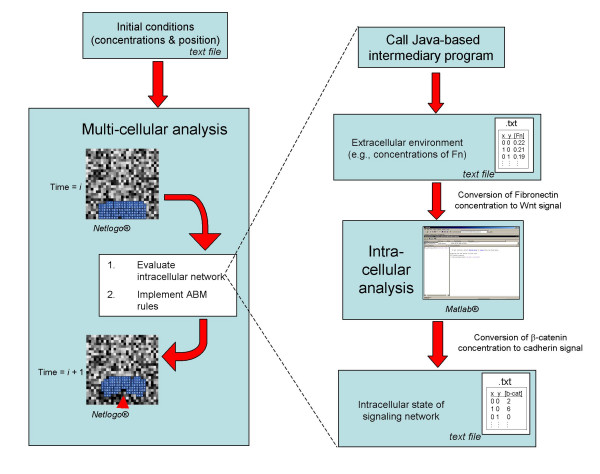
**Schematic depicting the multiscale computational framework**. The ABM outputs Fn levels at each pixel to a text file and opens a JAVA interface, which opens Matlab. Matlab imports the text file containing Fn concentrations, and the intracellular model calculates the amount of β-catenin signaling in each cell. These levels are input into the multi-cell model, and impact the extent of cadherin activation in each cell.

The multiscale simulation proceeds as follows (Figure 6). In the ABM, each cell is instructed to decide how to move based on the algorithm described above. Movement of cells results in remodeling of the Fn matrix. Each cell then calculates the average Fn concentration of the pixels it senses and reports its local Fn concentration to a text file. NetLogo then calls the JAVA intermediary, which calls the Matlab^® ^function. Next, Matlab^® ^runs the intracellular signaling pathway simulation (timestep = 3.5 minutes in the ABM simulation), which converts the Fn concentration experienced by each cell to a Wnt signal strength for each cell. There is evidence for a relationship between Wnt/β-catenin signaling and cadherin production, though the exact relationship is not explicitly defined [23]. The intracellular model then computes β-catenin concentrations based on Wnt signaling by analyzing the coupled ordinary differential equations (see Methods below), and exports β-catenin concentrations for each cell into a text file. After writing to the text file, the Matlab^® ^and JAVA intermediary programs close. The text file containing β-catenin concentrations is read by NetLogo so that each cell in the ABM is updated with a new cadherin concentration corresponding to the β-catenin concentration. NetLogo then repeats the cycle by initiating the movement algorithm. The final multiscale model was a fully automatic simulation that writes text files of Fn concentrations, β-catenin concentrations, and cell positions over time. In addition, the model writes a text file that summarizes the parameter values used in the simulation. The dynamics connecting Wnt signaling and cadherin production are simplified, and such parameters will need to be experimentally measured. However, for the purposes of the model discussed herein, this simplification was appropriate for illustrating the key features discussed below.

The intracellular and multicellular model operated on identical timescales: after the cells took a step that equated to 3.5 minutes of movement, the intracellular model ran for a simulation of 3.5 minutes. This enabled seamless temporal integration between the two biological scales. The goal of this early stage model was not the precise simulation of gastrulating tissue, but rather proof that the concepts of multiscale modeling can be effectively applied to capture multiscale phenomena (intracellular signaling and multicell interactions).

**Figure 7:**
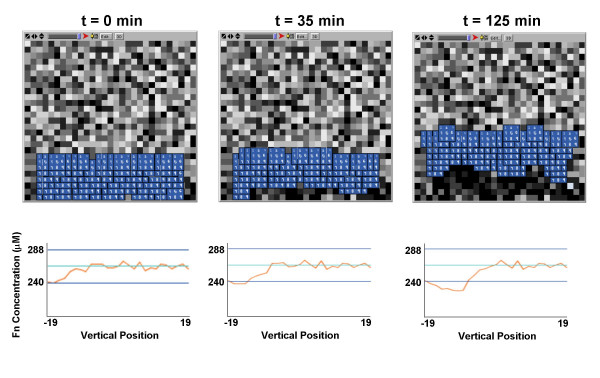
**Predictions of the multiscale model**. (Top Row) Screenshots from the multiscale model at different simulation time points show that cells migrate in a uniform direction while maintaining contact with one another. (Bottom Row) As cells migrate toward the top of the simulation space, a Fn matrix gradient is established from the leading edge to trailing edge of the mesendoderm explant, and this can also be seen in the darkening of pixels behind migrating cells.

Screenshots from three different time points of a representative multicellular simulation are shown in Figure 7 (**top row**). The multicellular model predicts a gradient of Fn underneath the explant, that increases from the trailing edge to leading edge and develops over time (Figure 7, **bottom row**). Moreover, the mesendodermal cells stay connected to one another as they migrate in a uniform direction. Neither of these emergent phenomena were programmed into the simulation, but emerged as the cells individually responded to the rule set and to their local environment. However, the rate of explant migration is reduced compared to the migration rate predicted by the ABM in the absence of the underpinning intracellular model (compare to Figure 3). This result is somewhat counterintuitive, but can be explained by the fact that the additional Wnt/β-catenin intracellular signaling pathway imparts an additional (negative) feedback loop that dictates the extent of cadherin activation, or cell-to-cell contact, based on local Fn concentrations. This additional potentiation of cadherin signaling leads to an overall reduction in cell-to-cell contacts, and causes an imbalance in the cadherin-to-integrin ratio, thus temporarily "locking" cells in place, and slowing the overall migration rate of the explant. This is evidenced by the fact that the cells are also more "spread out" in the multicellular simulation compared to the freestanding ABM.

## Conclusion

A framework for multiscale modeling of biological systems is presented. A published model of an intracellular signaling network key to morphogenesis was implemented. A novel multicellular ABM of mesendoderm migration was generated. Several hypothesized regulatory mechanisms were probed, including: (1) the role of cell-to-cell contacts mediated by cadherins in regulating the cohesiveness of the mesendoderm explant during development, (2) the role of the Fn matrix in modulating cell migration speed, (3) the necessity of a haptotactic polarity signal in regulating unidirectional migration of mesendodermal cells within the explant, and (4) the relationship between cadherins and integrins in promoting mesendoderm morphogenesis.

An intracellular model of Wnt/β-catenin signaling in the *Xenopus *embryo was implemented. This model was verified by reproducing the results presented in Lee et al. (2003). The model was modified to include user-defined inputs and outputs, so that it could read and write to text files that could be accessed by NetLogo. On the multicellular level, literature-supported rules were added to the model to direct cell movement and the program was modified to allow communication with JAVA and (indirectly) Matlab^®^. Aggregate cell movement was validated qualitatively for the multicellular scale model. Communication was achieved between the two separate models, resulting in a novel multiscale modeling approach.

The multiscale model retained specific characteristic behaviors of the observed phenomenon. One of the key outcomes of this project was the repeatability of the tissue movement. Each cell was allowed to randomly move and interact with other cells, as long as it obeyed simple rules. The randomness, however, consistently resulted in patterned behaviors, suggesting that inherent system characteristics, rather than individual agents, were responsible for the complex biological events. The model effectively represented the fibronectin attachments and shingling behaviors of cells, which resulted in the emergent phenomena of unified forward movement. When combined with the intracellular scale, the model gained sensitivity to changes within individual cells, which resulted in a more complete representation of the system.

Currently, the multiscale model is functional, but has many areas for improvement. There is insufficient quantitative data in literature relating the effects of Fn levels to the Wnt/β-catenin signaling pathway, and the effects of β-catenin levels to cadherin expression levels, so the linear relationships between these factors were qualitatively assumed. Determining these relationships will require additional experimental studies or may be inferred through a detailed parameter analysis of the simulation. Chemotactic gradients may also be important features of mesendoderm migration not yet included in the current model, and they will be critical features to consider in subsequent iterations (albeit the relative importance of chemotactic signals has been questioned and is a focus of current research [9]). Cell proliferation and death may also be considered in future model developments, although there is a lack of evidence of the relative importance of these processes as well. Computing limitations severely reduced the speed of the multiscale model. The code was designed to be easily expandable rather than computationally efficient, so the simulations required significant computational resources. Although this was not a problem in the abbreviated simulations, this could make a complete simulation of embryogenesis very time consuming. Finally, the connection of the intracellular signaling pathway to the multiscale model assumes that there are no changes during the 3.5 minute interval that the simulation is run. In reality, the pathway is constantly adapting to new conditions, and our model has no way to predict what happens within the 3.5 minute interval.

The models described herein set the stage for future improvements that will enhance predictive capabilities and enable their application to a wider range of biological questions. The intracellular level model can be adapted to include more signaling pathways, as mentioned above, including Wnt/JNK, PDGF, FGF and activin pathways, which have demonstrated importance in mesendoderm migration [20,24-27]. Moreover, the incorporation of a continuum-based model to approximate the actual forces experienced by cells as they migrate, exert traction forces on the Fn substrate, and push and pull neighboring cells is an important next step. The amount of mechanical force experienced by cells in this system is known to impact levels of integrin and cadherin activation, and therefore should be accounted for in this complex system. We suggest that this could be accomplished by developing a finite element model (FEM) that could also be implemented in Matlab^® ^and called by a JAVA program to communicate with NetLogo in a way that is similar, if not identical, to the intracellular signaling model described here. The integration of a FEM with a NetLogo-based ABM has already been accomplished to study the impact of fluid forces on white blood cell adhesion in the microcirculatory system [12].

Although there has been some work to develop multiscale computational analyses for various biological systems/processes [28-31], the immediate goal of this work is the creation of a "digital embryo." This will serve as a new tool for answering the larger questions in developmental biology that require more than the classical reductionist approach. Such models can become a community-wide effort to integrate data and hypotheses of key development processes. These computer simulations and models provide a forum for addressing competing hypotheses. In addition, there are differences between our proposed multiscale approach compared to that taken by others in this area. For example, we propose to utilize published parameters for the key signaling pathway and cell-level interactions. Others have arrived at systems level properties, such as robustness, by being more comprehensive in the total number of genes but by exclusively relying on parameter estimation [32]. Previous attempts at multiscale modeling in the vertebrate have not incorporated spatial and temporal systems-level heterogeneities, such as variations in the tissue environment and cell-to-cell differences in phenotype or genotype [33,34], although some have addressed this in invertebrates, such as *Drosophila *[35,36].

Since mesoderm migration depends on both intracellular and extracellular events, it was an ideal starting point for such analysis. In a recent review by [37] the need for a multiscale modeling framework is described in detail, as are the challenges faced by the computational physiology community. Despite the great strides made by the international "Physiome Project" in establishing computational models at each level of structural organization in a variety of organ systems, a central remaining challenge is to develop integrative frameworks that couple the single cell gene network to the multicell, heterogeneous environment. Our computational framework is a first step at modeling multiscale systems with molecular details for each cell and by incorporating spatial and temporal heterogeneities in an experimentally verifiable system.

## Methods

### Model system: *Xenopus laevis *gastrulation

*Xenopus laevis*, commonly known as the African clawed frog, is frequently used to study tissue morphogenesis during embryonic development because of its similarities to the same process in humans, and because its tissues can be surgically excised at different stages of embryogenesis and studied *ex vivo *by plating the explants on glass coverslips [5]. The modeling effort herein is, therefore, focused on the *ex vivo *migration of mesendodermal cells, which closely recapitulates their morphogenic behaviors *in vivo *between developmental stages 10 and 12 (Figure 1) [5].

### Wnt/β-catenin intracellular signaling network

A *Xenopus*-specific Wnt/β-catenin signaling pathway model was published by Lee et al. (2003) [38]. The authors primarily modeled this pathway with mass action kinetics. We reconstructed this model in Matlab^® ^(see Additional File 1). In addition, we created graphical user interfaces (GUIs) that enabled manipulation of the initial conditions for the system of ordinary differential equations for testing and debugging purposes. As appropriate, the default parameters (e.g., initial concentrations and rate constants) were set to the values published by Lee et al. (2003).

Our Matlab^® ^implementation of the model by Lee et al., 2003 was first validated by comparing our Matlab^® ^predictions to their published results. For example, steady state values were confirmed and all plots characterizing β-catenin levels over time with various perturbations to the network (e.g., gene knockout, overexpression) were reproduced. This validation process ensured that the system of ordinary differential equations was reconstructed correctly.

### Agent-based modeling (ABM) for multi-cellular simulations

ABM is a discrete modeling approach that enables the simulation of the behaviors of individual agents, or in this case, cells and their sub-cellular compartments, within a heterogeneous environment over time. ABM is particularly useful for studying emergent biological tissue patterning events, such as morphogenesis [13,14], where aggregate cell behaviors acting and interacting in space and time give rise to new tissue structures. Rules that govern agent (cell) behaviors are derived from the published literature and from independent experiments. The collection of rules, or a rule-set, dictates how an individual agent will act given different environmental stimuli, such as the presence of Fn at its discrete location or the presence of a neighboring cell in direct contact with it via cadherin coupling. The ABM, therefore, represents the collection of individual agent behaviors that emerge as they individually interact with one another and with their dynamic environment. ABM has been used to examine the morphogenic process of blastocoel roof thinning, another integral part of embryogenesis, thus substantiating the feasibility of applying ABM to the study of embryonic processes [13]. NetLogo, a software package available for free download [39], was used for the ABM in this study. The relevant Matlab, NetLogo, and Java code can also be downloaded from our lab websites (see [40] and [41]).

## List of abbreviations

ABM – Agent-based model(ing)

ECM – extracellular matrix

Fn – fibronectin

## Authors' contributions

SHR and CKS developed the agent-based model. ALL, SEM, and MAO implemented the intracellular signaling model. KRJ and BD helped generate the rules for the agent-based model. JAP, SMP, and DWD conceived of the project. SHR, CKS, ALL, SEM, DWD, JAP, and SMP wrote the paper.

## Supplementary Material

Additional file 1 multicellular_scale_model.nlogo. Multicellular model of *Xenopus *mesendoderm migration written in the NetLogo environment. Click here for file

Additional file 2 java_intermediary_code.doc. JAVA code for the program that integrates the Matlab and NetLogo models of *Xenopus *mesendoderm migration. Click here for file

Additional file 3 intracellular_scale_model.m. ODE-based model of Wnt/β-catenin signaling network, from Lee *et al*. PLoS Biol. 2003. Click here for file
