# Supplementary material for: Multiscale computational analysis of Xenopus laevis morphogenesis reveals key insights of systems-level behavior
Source: BMC Syst Biol. 2007 Oct 22;1:46. doi: 10.1186/1752-0509-1-46 (PMC2190763; doi:10.1186/1752-0509-1-46)
Supplement: Additional file 2 — java_intermediary_code.doc. JAVA code for the program that integrates the Matlab and NetLogo models of Xenopus mesendoderm migration. [file 1752-0509-1-46-S2.doc]

**Supplementary Data – JAVA code for integration program**

**ExecuteMatlab.java**

import org.nlogo.api.*;

public class ExecuteMatlab extends DefaultCommand

{

public String getAgentClassString() {

return "O";

}

public Syntax getSyntax() {

return Syntax.commandSyntax(new int[] {Syntax.TYPE_STRING});

}

public void perform(Argument[] args, Context context) throws ExtensionException {

String command = args[0].getString();

System.out.println("Extension Running");

System.out.println(command);

try {

Runtime runtimeobj = java.lang.Runtime.getRuntime();

Process mlab = java.lang.Runtime.getRuntime().exec("matlab " + command); // try executing it

try {

mlab.waitFor();

} catch (java.lang.InterruptedException ex) {

ex.printStackTrace();

throw new ExtensionException("Command generated an interruption exception");

}

} catch (java.io.IOException ioex) {

ioex.printStackTrace();

throw new ExtensionException("Command generated an IOException");

}

System.out.println("Extension Finished");

}

}

**ExecuteMatlabManager.java**

import org.nlogo.api.*;

public class ExecuteMatlabManager extends DefaultClassManager {

public void load(PrimitiveManager primitiveManager) {

primitiveManager.addPrimitive("execute-in-matlab", new ExecuteMatlab());

}

}
